# Cost-minimisation analysis of oritavancin for the treatment of acute bacterial skin and skin structure infections from a United Kingdom perspective

**DOI:** 10.1007/s10198-022-01432-2

**Published:** 2022-02-03

**Authors:** Daniela Zinzi, Ioanna Vlachaki, Edel Falla, Theo Mantopoulos, Dilip Nathwani

**Affiliations:** 1 Menarini Ricerche Spa, Florence, Italy; 2 EMEA Real World Methods and Evidence Generation, IQVIA Ltd, London, UK; 3 EMEA Real World Methods and Evidence Generation, IQVIA Ltd, Athens, Greece; 4 Medical School, University of Dundee, Dundee, DD19SY UK

**Keywords:** Acute bacterial skin and skin structure infections, United Kingdom, Oritavancin, Cost-minimisation analysis, Methicillin-resistant *Staphylococcus aureus* infection, I10, I18

## Abstract

**Background:**

Early discharge (ED) from hospital and outpatient parenteral antibiotic therapy (OPAT) are effective approaches for the management of a range of infections, including acute bacterial skin and skin structure infections (ABSSSI). Strategies that facilitate ED, thereby reducing complications such as healthcare-acquired infection whilst enhancing patient quality of life, are being increasingly adopted in line with good antimicrobial stewardship practice. This study presents a cost-minimisation analysis for the use of oritavancin at ED versus relevant comparators from a National Health Service (NHS) and personal and social services United Kingdom perspective.

**Methods:**

A cost-minimisation model considering adult patients with ABSSSI with suspected or confirmed methicillin-resistant *Staphylococcus aureus* (MRSA) infection, was developed based on publicly available NHS costs, practice guidelines for ABSSSI and clinical expert’s opinion. Cost of treatment and treatment days were compared for oritavancin at ED to dalbavancin, teicoplanin, daptomycin and linezolid.

**Results:**

Following the empiric use of either flucloxacillin or vancomycin in the inpatient setting, oritavancin was compared to OPAT with dalbavancin, teicoplanin and daptomycin, and oral linezolid from day 4 of treatment. Oritavancin at ED reduced treatment duration by 0.8 days and led to cost savings of £281 in comparison to dalbavancin. In comparison to teicoplanin, daptomycin and linezolid, oritavancin reduced treatment duration by 5 days, with marginally higher costs (£446, £137, and £1,434, respectively).

**Conclusion:**

Oritavancin, used to support ED, is associated with lower costs compared with dalbavancin and reduced treatment duration relative to all comparators. Its use would support an ED approach in MRSA ABSSSI management.

## Introduction

Complicated skin and skin structure infections (cSSSIs) represent acute microbial invasions of the skin and underlying soft tissues and pose considerable health burden that result in hospitalisation, surgical procedures, complications such as bacteraemia and life-threatening necrotising infections [1, 2]. In 2013, cSSSIs were redefined by the United States Food and Drug Administration as acute bacterial skin and skin structure infections (ABSSSI) [3]. Under this new definition, ABSSSI includes cellulitis/erysipelas, wound infections and major cutaneous abscesses with a minimum lesion surface area of approximately 75 cm^2^ [3]. Causative ABSSSI pathogens are mostly gram-positive with *Staphylococcus aureus,* including methicillin-resistant *Staphylococcus aureus* (MRSA), *and Streptococcus pyogenes* strains representing the most prevalent pathogens [3, 4]. In accordance with regulatory agencies’ definitions, the study population for ABSSSI and cSSSIs may vary; however, in routine clinical practice both represent severe skin and skin structure infections [3, 5].

The emergence of MRSA infections, with high pathogenicity and multi-drug resistance has altered the paradigm of patients at risk of such infections due to reduced effective treatment options. Although the European Union and European Economic Area population-weighted mean MRSA percentage has decreased from 19.6% in 2014 to 16.4% in 2018, with a large variability in country-specific rates, MRSA continues to impose a considerable challenge in the management of ABSSSI [6, 7]. Hospital Episode Statistics trends in England from 1991 to 2006 show an increase in hospitalisations and general practice prescriptions for staphylococcal infections [8]. A point prevalence survey conducted in 2012 across 99 National Health Service (NHS) acute trusts reported that 15.7% of the healthcare associated infections (HCAIs) were ABSSSI [9]. As such in 2012, the United Kingdom (UK) government adopted a zero-tolerance approach for avoidable HCAIs with an emphasis on MRSA infections [10]. Early discharge (ED) of suitable patients with appropriate support has a potential to reduce HCAIs [11].

Antibacterial agents such as beta-lactams, macrolides, lincosamides and fluoroquinolones are frequently used for the treatment of ABSSSI and aim to minimise tissue damage and prevent the spread of infection [3, 5, 12–14]. A pan-European study conducted in 2010–2011 involving 1542 hospitalised patients with MRSA cSSSI found that the most frequently used first-line antibiotics were vancomycin (50.2%), linezolid (15.1%), clindamycin (10.8%) and teicoplanin (10.4%), whilst linezolid was the most preferred drug during discharge [15].

ABSSSI can result in severe complications imposing significant morbidity and a high economic burden on the healthcare systems due to the associated hospitalisation and healthcare costs [8, 16, 17]. For example, a retrospective observational study from 12 European countries to evaluate treatment patterns of patients with MRSA cSSSI estimated the length of intravenous (IV) antibiotic therapy as 10.1 days in the UK, with hospital length of stay ranging from 15.2 to 25.0 days across Europe [15]. In another observational European cohort study looking at morbidity and practice, 1,995 patients were hospitalised due to cSSSI, whilst more than half of the study participants were admitted to intensive care unit admissions (56.2%), and experienced bacteraemia (51.6%), with a mortality rate of 3.4% [18]. However, there are limited studies highlighting the true cost of ABSSSI to the NHS UK.

The treatment course for ABSSSI ranges from 5 to 14 days of systemic IV antibiotics. However, with the availability of outpatient parenteral antibiotic therapy (OPAT), the inpatient treatment duration can be reduced by prescribing IV antibiotics at discharge [15, 19]. Switching to OPAT/oral therapy can potentially reduce the total healthcare cost of treatment for patients with ABSSSI [20]. However, OPAT requires supplementary resources and is not a feasible alternative for all patients [21, 22]. Since many ABSSSI necessitate inpatient treatment, developing and implementing early switch to oral/OPAT and ED have the potential to decrease NHS costs and resources, if deemed clinically appropriate. The adoption of ED has been facilitated by the introduction of long-acting antibiotics such as oritavancin and dalbavancin that can be administered as a single dose without the need for OPAT [15, 23–25].

Oritavancin, a second-generation lipoglycopeptide derived from chloro-eremomycin, was approved by the European Medicines Agency for the treatment of ABSSSI caused by susceptible gram-positive bacteria including MRSA in 2015 [26]. It demonstrated non-inferiority to vancomycin for the treatment of ABSSSI in the global phase III randomised clinical trials- SOLO I and SOLO II [27, 28].

To our knowledge, the economic impact of the adoption of oritavancin at ED versus the current standard of care (SoC) has not been investigated for the treatment of ABSSSI from an NHS and personal and social services (PSS) UK perspective. This study aims to report the outcomes from a cost-minimisation evaluation of oritavancin at the time of ED versus the current SoC, in patients with confirmed or suspected MRSA infections from the perspective of the NHS/PSS UK.

## Methods

### Model structure

**Fig. 1:**
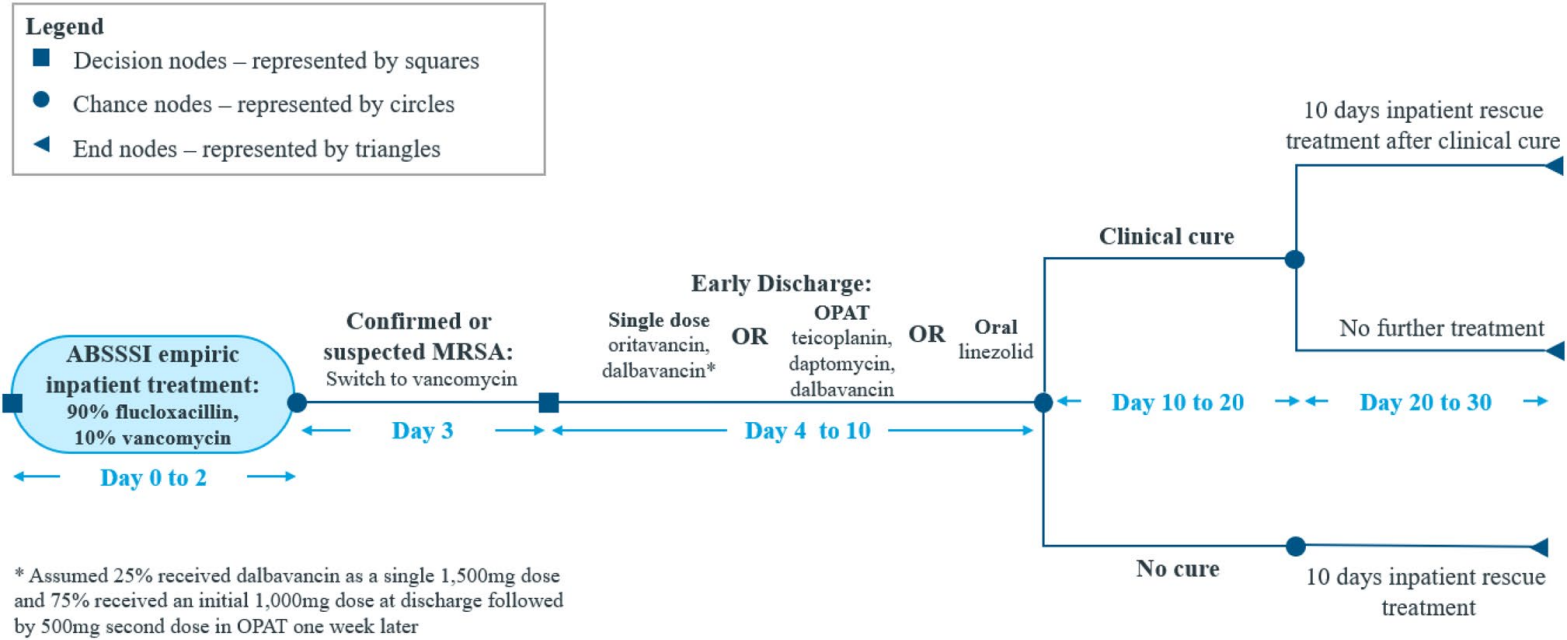
Cost-minimisation model. *ABSSSI* Acute bacterial skin and skin structure infections, *MRSA* Methicillin-resistant *Staphylococcus aureus*, *OPAT* Outpatient parenteral antimicrobial therapy

A cost-minimisation model (CMM) (Fig. 1) was developed in Microsoft Excel® from an NHS/PSS UK perspective. The time horizon of the model was 30 days and included the initial treatment course, second-line treatment, follow-up, and rehospitalisation. The interventions considered in the CMM were oritavancin in comparison to dalbavancin, OPAT teicoplanin, OPAT daptomycin, and oral linezolid at ED. The model did not account for the cost of MRSA laboratory testing (culture or polymerase chain reaction [PCR]-based testing), as both the intervention and the comparator arms in the model were subject to this cost and the overall impact in the incremental results was zero.

The model was designed to closely reflect UK clinical practice. To ensure this, the model structure, clinical and cost inputs, time horizon, positioning and associated assumptions were validated by a clinical expert, also a co-author in the present analysis. All patients with ABSSSI included in the analysis were initiated on treatment with empiric therapy on day 0 to day 2 with either flucloxacillin (90% patients) or vancomycin (10% patients) as inpatient treatment. On day 3, it was assumed that 100% of the patients had confirmed MRSA infection and were switched to vancomycin. On day 4, it was assumed that 100% patients were eligible for ED to outpatient treatment with the following therapeutic treatment options: single dose IV oritavancin, dalbavancin (either as a single 1500 mg dose or two doses: 1000 mg initial dose, followed by 500 mg a week later), OPAT teicoplanin, OPAT daptomycin, or oral linezolid until day 10. On day 10, all cured OPAT patients were discharged from clinical care. However, if clinical cure was not confirmed, patients were deemed as treatment failures and a 10-day course of inpatient rescue therapy with IV linezolid was initiated. It was also assumed that a subset of patients clinically cured after first-line therapy may experience relapse requiring hospitalisation, these relapses were associated with a 10-day inpatient rescue therapy.

### Model inputs and data sources

#### Population inputs

The target population includes adult patients (≥ 18 years of age) with ABSSSI with suspected or confirmed MRSA infection who are eligible for ED in the NHS UK. Mortality was not considered in the model due to the short time horizon and statistically similar mortality rates between interventions according to the literature and clinical expert opinion [29].

#### Clinical efficacy inputs

A cost-minimisation approach, which assumed equivalent efficacy for oritavancin and the model comparators, was adopted for this analysis. This was supported by recent network meta-analysis, which found no statistically significant differences in efficacy for oritavancin versus all SoC comparators in the intent-to-treat population and for oritavancin versus daptomycin and linezolid in the MRSA population, [29], as comparative evidence against teicoplanin and dalbavancin was not available in the MRSA population. This was also validated by the clinical expert.

For oritavancin and vancomycin, the clinical cure rates were taken from the pooled SOLO I [27] and SOLO II [28] clinical trials. As the trials demonstrated that the two treatments were statistically equivalent, the trial data were combined across treatment arms to form a common cure rate. The cure rate for oritavancin and vancomycin in the MRSA population was 83.7% (Table 1). As teicoplanin, daptomycin, linezolid and dalbavancin were all considered as therapies given at the time of ED following initial inpatient treatment with vancomycin, separate cure rates were not considered. The rehospitalisation rate, following clinical cure/failure at day 10 was assumed as 3.8% based on a study by Marwick et al. [30] based on clinical expert opinion (Table 1).

**Table 1:** Clinical efficacy inputs

Parameter	Value	Source
Confirmed MRSA, *n* (%)	169 (82.8)	SOLO I [27]
	170 (84.6)	SOLO II [28]
	339 (83.7)	SOLO pooled
Rehospitalisation after clinical cure assessment, %	3.8	Marwick et al. [30]

*MRSA* Methicillin−resistant *Staphylococcus aureus*

#### Cost inputs

Antibacterial medication costs were obtained from the British National Formulary (BNF) 2020 (Table 2) [31]. Resource use costs were obtained from the literature and publicly available sources. Drug prices were obtained from the BNF; for treatments with multiple generic options, the lowest cost option (per vial) was used. As per the clinical expert opinion it was assumed that for dalbavancin dose, 25% patients received dalbavancin as a single 1500 mg dose, whereas 75% of patients received two doses; 1000 mg initial dose, followed by 500 mg a week later. The cost per day of inpatient stay was based on the average cost of soft-tissue disorders derived from the latest NHS costs of admitted patient care and outpatient procedure prices (Table 2). The cost of treatment also included the laboratory test costs (vancomycin therapeutic drug monitoring) and ambulatory costs (physician visit and OPAT or clinic infusion), obtained from published sources and Unit Costs of Health and Social Care-Personal Social Services Research Unit (PSSRU) 2019 (Table 2) [20, 32–35]. All costs were reported in GBP (£) and where necessary, were inflated to 2020 using the NHS cost inflation index, PSSRU 2019 [32–34].

**Table 2:** Antibacterial dosing costs and costs for treatment

Cost category	Daily dosage*	Daily cost (£)	Source(s)
Medication daily costs			
Oritavancin (single dose cost)	1200 mg single dose	1500	Menarini 2020 [50]
Flucloxacillin (IV)	1 g qid	14	BNF 2020 [31]
Vancomycin (IV)	1143 mg (15 mg/kg) bid	26	
Linezolid (IV)	600 mg bid	89	
Linezolid (oral)	600 mg bid	16	
Dalbavancin (IV)	1500 mg**	1676	
Daptomycin (IV)	350 mg od	60	
Teicoplanin (IV)	400 mg bid on day 1, 400 mg od subsequently	7	
Hospitalisation daily costs			
Inpatient stay	Per day	777	NHS 2019/20 National Tariff Payment System- Average cost of soft-tissue disorders calculated based on Admitted patient care and outpatient procedure prices [51]
Laboratory test costs			
Vancomycin TDM (per day)	Per day	0.9	Seaton et al. [20]
Ambulatory costs			
Physician visit	Per visit	38	Unit costs of health and social care (PSSRU) 2019 [32–34]
OPAT or clinic infusion	Per infusion	173	National Schedule of NHS costs 2018–2019—average cost of all outpatient appointment for adults from Outpatient Attendances Data (£169) [35]

*Bid* twice a day, *BNF* British National Formulary, *IV* Intravenous, *NHS* National Health Service, od once daily, *OPAT* Outpatient parenteral antibiotic therapy, *PSSRU* Personal Social Services Research Unit, *qid* four times a day, *TDM* Therapeutic drug monitoring

*The daily dosage was weight-based, the weight was assumed as equal to 76.2 kg, which is the average weight of the oritavancin arm of the SOLO II trial [27]

**Assumed that 25% patients receive Dalbavancin as a single 1500 mg dose and 75% of patients receive a dose of 1000 mg followed by 500 mg one week later

#### Treatment days and outpatient/OPAT inputs

Outpatient oral and OPAT resource use were dependent upon route of administration and were validated by the clinical expert (Table 3). Treatment days were defined as the number of days during which a patient actively received treatment thus, a course of oritavancin is associated with one treatment day, as it is administered as a single dose. The frequency of OPAT sessions per day for daptomycin and teicoplanin was 1, whereas for dalbavancin it was 0.13 (i.e., 1 visit per duration of OPAT), allowing for 75% of the patients to receive a second dalbavancin dose of 500 mg as OPAT (Table 3) based on clinical expert opinion.

**Table 3:** Resource utilisation inputs in outpatient setting

Frequency	Treatment arm	Resource use per day	References
OPAT sessions per day	All oral treatments	0	Based on clinical expert opinion
	Oritavancin	0	
	Dalbavancin	0.13*	
	All IV OPAT	1	
Outpatient visits per day (discharge → cure)	All oral treatments	0	
	Oritavancin	0.14**	
	Dalbavancin	0.04***	
	All IV OPAT	0	
Outpatient visits per day (cure → rehospitalisation)	All	0	

*OPAT* Outpatient parenteral antibiotic therapy

*Assuming that 75% of patients receive their second 500 mg of dalbavancin as OPAT

**One outpatient visit per week

***Assuming that 25% of patients who have 1500 mg single dose attend a weekly follow-up appointment

The frequency of outpatient visits per day, from discharge to cure for oritavancin was 0.14 (i.e., 1 visit per week), whereas for dalbavancin it was 0.04 (i.e., 1 visit per week), assuming that 25% of the patients received single 1,500 mg dalbavancin dose (Table 3).

### Sensitivity analysis

One-way sensitivity analysis (OWSA) was conducted for all clinical and cost parameters by investigating the outcomes around the upper and lower 30% variance of input parameters. Scenario analysis was also conducted for the following parameters by investigating plausible values from the reported outcomes: (a) days until considered for outpatient discharge; (b) proportion of patients starting on flucloxacillin suitable for ED; (c) proportion of patients in oritavancin treatment arm requiring rehospitalisation; (d) proportion of patients starting empirical therapy with vancomycin; and (e) days until pathogen confirmation. The effect of the scenarios above are presented in Tornado diagrams. Probabilistic sensitivity analysis (PSA) was conducted for incremental costs, incremental treatment days and incremental inpatient days using 1000 Monte-Carlo iterations. PSA results are presented in the form of scatterplots showing total cost versus treatment days.

## Results

### Base case results

The cost breakdown and cost-minimisation results for oritavancin at ED with relevant comparators are presented in Table 4. Total costs include medication costs, inpatient costs (initial treatment course, post-treatment failure, rehospitalisation), OPAT costs, and outpatient costs. The total medication cost- for oritavancin (£1751) was lower in comparison to dalbavancin (£1927), whilst it was higher than teicoplanin (£302), daptomycin (£611), and linezolid (£349). The inpatient costs were the same across all comparators (£3326). Teicoplanin, daptomycin, and dalbavancin were associated with OPAT for IV administration (£1035, £1035, and £129, respectively). OPAT costs for dalbavancin were lower than teicoplanin and daptomycin as only 75% of patients received their second dose of dalbavancin as OPAT (25% of patients received dalbavancin as a 1500 mg single dose). Oritavancin and dalbavancin were associated with outpatient costs for monitoring/ follow-up (£ 32 and £ 8, respectively). Oritavancin was associated with cost savings in comparison to dalbavancin (£281) and higher incremental costs in comparison to teicoplanin (£446), daptomycin (£137) and linezolid (£1434).

**Table 4:** Cost breakdown and cost-minimisation results for oritavancin at early discharge

Outcome	Oritavancin	Teicoplanin	Daptomycin	Linezolid	Dalbavancin
Total costs (£)*	5020	4573	4882	3586	5301
Total medication costs (£)*	1751	302	611	349	1927
Total inpatient costs (£)*	3236	3236	3236	3236	3236
OPAT costs (£)*	0	1035	1035	0	129
Outpatient costs (£)*	32	0	0	0	8
Mean treatment days	7.0	12.0	12.0	12.0	7.8
Mean inpatient days	6.0	6.0	6.0	6.0	6.0
Difference in costs (£)	–	446**	137**	1434	− 281
Difference in treatment days	–	− 5.0	− 5.0	− 5.0	− 0.8
Difference in inpatient days	–	0.0	0.0	0.0	0.0
Cost per treatment day avoided (£)	–	89	27	287	Oritavancin at ED is dominant

*ED* early discharge

*All costs are rounded to the nearest GBP (£)

**The difference in costs do not sum up due to rounding up of decimal values

With respect to treatment duration, oritavancin was associated with a reduction in treatment days versus all comparators, ranging from 0.8 (versus dalbavancin) to 5.0 days (versus teicoplanin, daptomycin and linezolid).

Oritavancin was the dominant comparator in contrast to dalbavancin, with reduced costs and treatment days. Whereas, considering the reduction in treatment days, it was estimated that oritavancin was associated with a small incremental cost per treatment day avoided in comparison to teicoplanin, daptomycin and linezolid (£89, £27, £287, respectively).

### Sensitivity analysis results

**Fig. 2:**
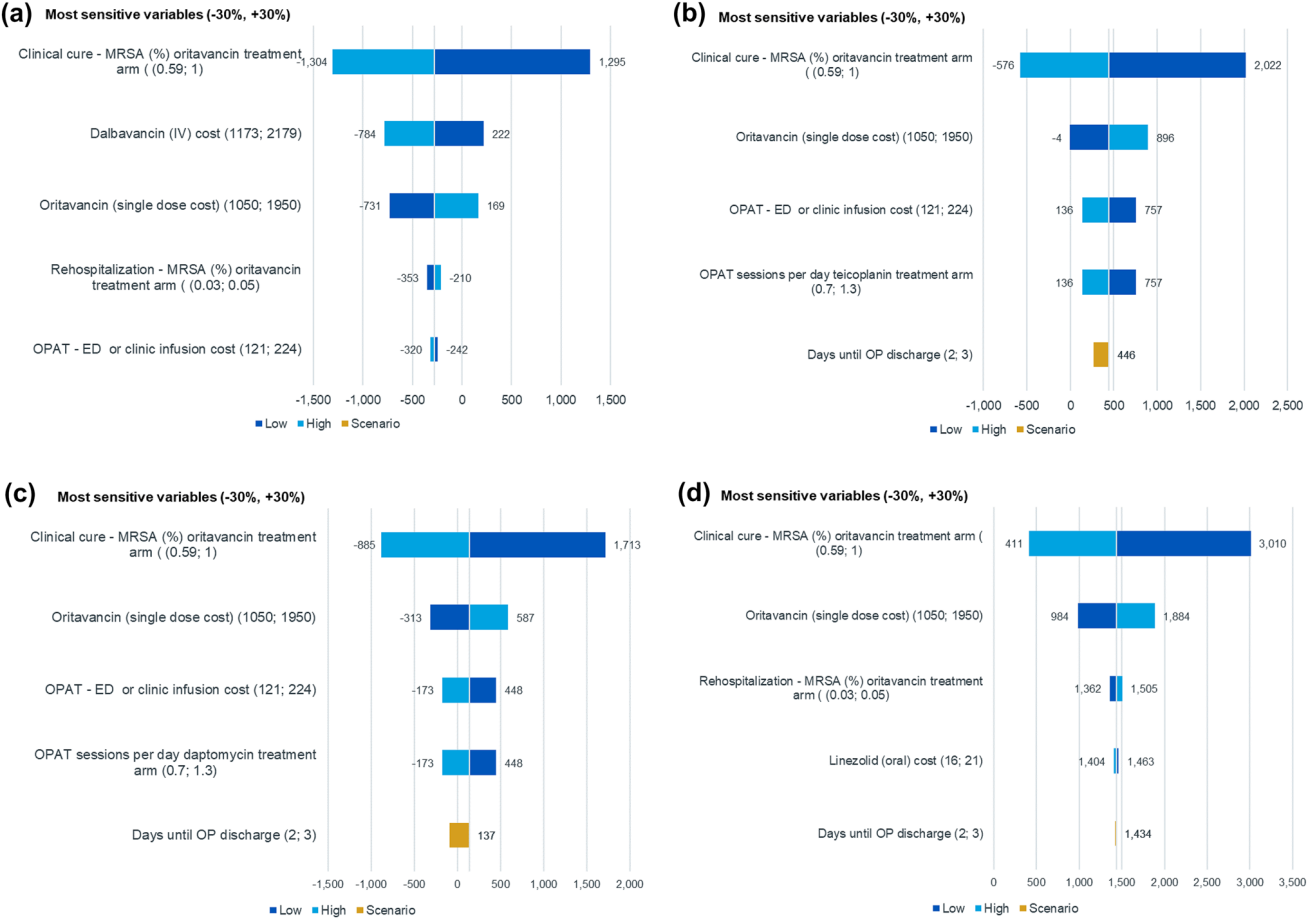
**a** Tornado diagram: Oritavancin versus Dalbavancin. *ED* early discharge, *IV* intravenous, *MRSA* Methicillin-resistant *Staphylococcus aureus*, *OPAT* Outpatient parenteral antibiotic therapy, *OWSA* One-way sensitivity analysis. **b** Tornado diagram: Oritavancin versus Teicoplanin. *ED* Early discharge, *MRSA* Methicillin-resistant *Staphylococcus aureus*, *OP* Outpatient, *OPAT* Outpatient parenteral antibiotic therapy, *OWSA* One-way sensitivity analysis. **c** Tornado diagram: Oritavancin versus Daptomycin. *ED* Early discharge, *MRSA* Methicillin-resistant *Staphylococcus aureus*, *OP* Outpatient, *OPAT* Outpatient parenteral antibiotic therapy, *OWSA* One-way sensitivity analysis. **d** Tornado diagram: Oritavancin versus linezolid. MRSA Methicillin-resistant *Staphylococcus aureus*, *OP* Outpatient, *OWSA* One-way sensitivity analysis

The OWSA results for incremental costs between oritavancin and dalbavancin, teicoplanin, daptomycin and linezolid are presented in Fig. 2a–d. These indicate that the base case is most sensitive to the cure rate of oritavancin and the cost of dalbavancin. Rehospitalisation was the fourth most important driver of costs versus dalbavancin (Fig. 2a) and third most important driver of costs versus linezolid (Fig. 2d). Other clinical and cost parameters had a negligible impact on the incremental cost.

**Fig. 3:**
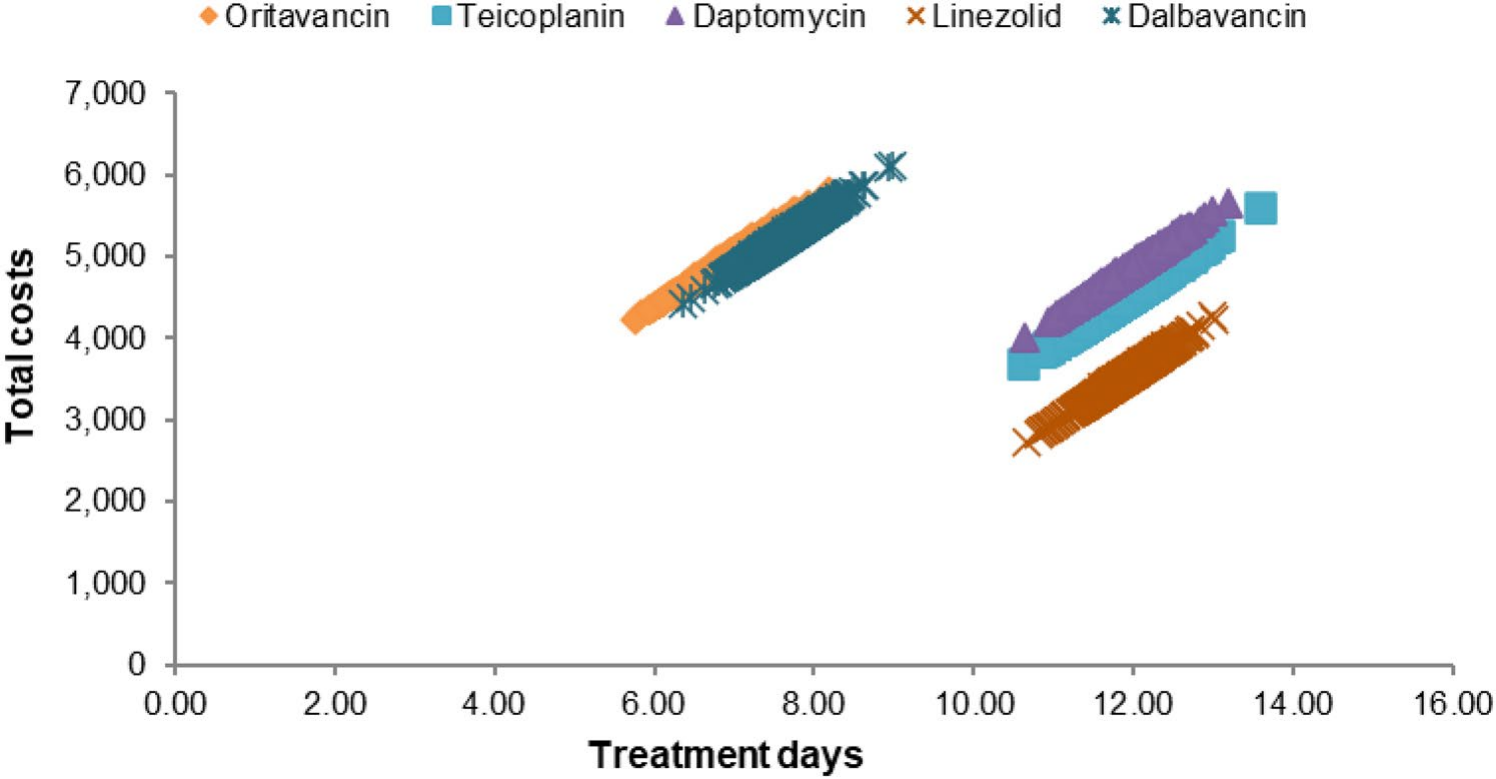
Scatterplot of total costs versus treatment days for oritavancin and comparators

The PSA results comparing total costs and treatment days for oritavancin versus all comparators are presented in Fig. 3. The mean costs for oritavancin, teicoplanin, daptomycin, linezolid and dalbavancin were £4974, £4529, £4833, £3537, and £5251, respectively. The mean treatment duration for oritavancin was 7 days and that for dalbavancin was 8 days. The mean treatment duration for teicoplanin, daptomycin and linezolid was 12 days each. The difference in mean treatment duration between the interventions is attributed to the shorter duration of treatment for oritavancin (1 day) and dalbavancin (75% 2 days) compared to teicoplanin and daptomycin (6 days) (Table 5). These results indicate the robustness of the analysis to input parameter variation, given that the probabilistic estimates are in close agreement to the deterministic ones.

**Table 5:** Treatment duration

Treatment	Duration	References
Empiric treatment (flucloxacillin, vancomycin)	4 days	Antibiotic steering committee, Salford Royal NHS Foundation Trust 2014 [52]
Outpatient treatment (OPAT daptomycin, OPAT teicoplanin/oral linezolid)	Additional 6 days	
Dalbavancin	Additional 2 days (75 of patients), additional 1 day (25% of patients)	[36]
Oritavancin	Additional 1 day	[24]
Second line/rescue therapy	Additional 10 days	Eckman et al*.* [15]

*OPAT* outpatient parenteral therapy

## Discussion

The cost-minimisation analysis assesses the potential cost savings associated with the use of oritavancin at the time of ED, compared to SoC for ABSSSI with suspected or confirmed MRSA infection, from an NHS/PSS UK perspective. The results of our analysis suggest that treatment with oritavancin results in lower total costs and fewer treatment days when compared to dalbavancin. The main driver for the higher total costs with dalbavancin is due to the higher medication cost of dalbavancin [36].

Oritavancin use at the time of ED results in a reduction in treatment days when compared to teicoplanin, daptomycin and linezolid. Although the total cost of treatment with oritavancin was higher when compared to these comparators, this was associated with a relatively marginal cost per treatment day avoided. The OWSA and PSA results confirm the overall robustness of the base case results to parameter variation.

The emerging literature suggests that shifting ABSSSI treatment to the outpatient settings results in cost savings and that the single dose treatment may be the preferred treatment option among a group of patients [25]. For example, in a study assessing the economic value of shifting ABSSSI care to outpatient settings from the US payer perspective using a literature-based decision tree economic model, the medical cost savings were estimated to be greater than 53% in the avoidable hospital admission scenario. Outpatient scenarios represented about 14–37% of total medical cost, with PICC accounting for 28–43% of the outpatient burden [37]. A survey conducted across 6 US hospital involving 94 patient responses, assessing patient preferences for treatment disposition and antibiotic therapy of ABSSSI in the emergency department indicated that outpatient care and single dose antibiotics are the preferred choice for ABSSSI treatment from the patient’s perspective as well [38]. In a Delphi survey involving 57 infectious disease specialists and microbiologists from Europe, 100% respondents concurred that long-acting antibiotics can be useful in antimicrobial stewardship programmes in ABSSSI as they improve patient’s compliance and reduce the length of hospitalisation [39]. In a US retrospective study, involving 75 patients that received oritavancin between 2015 and 2017, the rationale given by clinicians for the use of oritavancin in most patients was the avoidance of long-term IV treatment (61%). Over half (57%) of the patients in this study avoided at least one hospital day with inpatient oritavancin treatment, resulting in cost savings of US $343,654 [40]. Length of hospital stay has been accredited as a substantial factor in driving health care costs for ABSSSI with MRSA [41]. Oritavancin provides a single dose treatment for ABSSSI with reduced costs of care through the elimination of multiple infusions of antibiotic therapy [42].

Our analysis is consistent with previous economic studies from the US and other parts of Europe comparing treatment strategies and therapeutic options in ABSSSI care. A US retrospective, observational cohort analysis comparing 30-day hospital admission rates and mean healthcare costs among 6,815 patients with ABSSSI, who received outpatient oritavancin and vancomycin in patients using a health market scan database over a 1-year time period, showed significantly lower 30-day admission rate with oritavancin versus vancomycin (6.1% versus 16.2%) [43], consistent with the results of this oritavancin model. Results from this oritavancin study are also in line with a CMM comparing the costs of inpatient vancomycin versus outpatient oritavancin treatment of patients with ABSSSI from a premier research database in the US, where switching from inpatient vancomycin treatment to outpatient oritavancin treatment estimated to save $1752.46 to $6475.87 per patient [44]. In another US hospital-based budget impact analysis involving 1000 patients diagnosed with ABSSSI receiving IV MRSA antibiotics over a 1-year time horizon, the use of oritavancin resulted in a reduction of total annual budget by 12.9% (US$1.23 million, approximately US$1234.67 per patient) [44]. In Europe, an analysis conducted in NHS Greece, Italy and Spain to assess the economic consequences of adopting ED strategy for the treatment of ABSSSI estimated a major reduction in total number of hospitalisation days (32–41%) and total healthcare costs (30–42%) [45], consistent with the results of this oritavancin study.

To simulate the complexity of the decision-making process treatment of ABSSSI, this analysis relied on several assumptions. The simulation was challenging due to variation of practice across hospitals and countries. The number of patients eligible for ED and treatment decision-making was based on clinical expert opinion due to a lack of published data. All individuals were initially treated empirically with IV flucloxacillin (90%) or IV vancomycin (10%) in the analysis. For dalbavancin treatment, patients were distributed as 25% single dose and 75% with two doses (second dose as OPAT) based on clinical expert opinion. Mortality and quality of life were not considered in the CMM due to the short time horizon of the CMM. Adverse events were not considered in the CMM, due to similar safety profiles between comparators [46–49]. Costs for the additional components needed for drug infusions (such as glucose and sodium chloride) were not considered. All the above assumptions were considered appropriate by the clinical expert.

## Conclusion

To our knowledge, this study is the first analysis comparing the healthcare costs of oritavancin versus SoC at ED for treatment of MRSA ABSSSI, from an NHS/PSS UK perspective. In comparison to dalbavancin, oritavancin reduced costs and treatment days for the treatment of patients with MRSA. As compared to teicoplanin, daptomycin and linezolid, oritavancin use at ED resulted in a reduction of five treatment days with marginal cost per treatment day avoided. Our findings indicate that the use of oritavancin has the potential to facilitate ED for ABSSSI, resulting in reduced treatment days and cost savings for the NHS UK.

## Data Availability

All data generated or analysed during this study is included in this published article (and its supplementary information files).

## References

[1] Esposito S, Noviello S, Leone S (2016). Epidemiology and microbiology of skin and soft tissue infections. Curr. Opin. Infect. Dis..

[2] Pollack CV, Amin A, Ford WT, Finley R, Kaye KS, Nguyen HH (2015). Acute bacterial skin and skin structure infections (ABSSSI): practice guidelines for management and care transitions in the emergency department and hospital. J. Emerg. Med..

[3] USFDA. Guidance for industry acute bacterial skin and skin structure infections: developing drugs for treatment. Center for Drug Evaluation and Research (CDER), Food and Drug Administration https://www.fda.gov/media/71052/download (2013, accessed Jul 01, 2020)

[4] Tong SYC, Davis JS, Eichenberger E, Holland TL, Fowler VG (2015). *Staphylococcus aureus* infections: epidemiology pathophysiology. Clin. Manif. Manag..

[5] NHS. Skin and Soft Tissue Infections (SSTI) Antibiotic Guidelines (Adult), https://www.srft.nhs.uk/EasysiteWeb/getresource.axd?AssetID=8102&type=full&servicetype=Inline (2019, accessed Jul 24, 2020)

[6] Livermore DM, Mushtaq S, Warner M, James D, Kearns A, Woodford N (2015). Pathogens of skin and skin-structure infections in the UK and their susceptibility to antibiotics, including ceftaroline. J. Antimicrob. Chemother..

[7] ECDC. European Centre for Disease Prevention and Control; Surveillance of antimicrobial resistance in Europe 2018; Annual report of the European Antimicrobial Resistance Surveillance Network (EARS-Net), https://www.ecdc.europa.eu/sites/default/files/documents/surveillance-antimicrobial-resistance-Europe-2018.pdf (2018, accessed Aug 08, 2020)

[8] Hayward A, Knott F, Petersen I, Livermore DM, Duckworth G, Islam A (2008). Increasing hospitalizations and general practice prescriptions for community-onset staphylococcal disease England. Emerg. Infect. Dis..

[9] Health Protection Agency. (2012) English National Point Prevalence Survey on Healthcare Associated Infections and Antimicrobial Use, https://webarchive.nationalarchives.gov.uk/20140714095446/http://www.hpa.org.uk/webc/HPAwebFile/HPAweb_C/1317134304594 (2011, accessed 24 Jul 2020)

[10] Department of Health; Technical Guidance for the 2012/13 Operating Framework, https://assets.publishing.service.gov.uk/government/uploads/system/uploads/attachment_data/file/216413/dh_132045.pdf (2011, accessed 08 Aug 2020)

[11] Dryden M, Saeed K, Townsend R, Winnard C, Bourne S, Parker N (2012). Antibiotic stewardship and early discharge from hospital: impact of a structured approach to antimicrobial management. J. Antimicrob. Chemother..

[12] Stevens DL, Bisno AL, Chambers HF, Dellinger EP, Goldstein EJ, Gorbach SL (2014). Practice guidelines for the diagnosis and management of skin and soft tissue infections: 2014 update by the Infectious Diseases Society of America. Clin. Infect. Dis..

[13] Sartelli M, Guirao X, Hardcastle TC, Kluger Y, Boermeester MA, Raşa K (2018). 2018 WSES/SIS-E consensus conference: recommendations for the management of skin and soft-tissue infections. World J. Emerg. Surg..

[14] Fulton, R., Doherty, L., Gill, D.H.A., Harper, C., Jenkinson, H., Loughrey, A., Martin, E., Mullan, J., Mullan, C., Scott, M., Witherow, A.: Guidelines on the management of cellulitis in adults (CREST), https://www.rcem.ac.uk/docs/External%20Guidance/10n.%20Guidelines%20on%20the%20management%20of%20cellulitis%20in%20adults%20(CREST,%202005.pdf (2005, accessed 08 Jul 2020)

[15] Eckmann C, Lawson W, Nathwani D, Solem CT, Stephens JM, Macahilig C (2014). Antibiotic treatment patterns across Europe in patients with complicated skin and soft-tissue infections due to meticillin-resistant *Staphylococcus aureus*: a plea for implementation of early switch and early discharge criteria. Int. J. Antimicrob. Agents..

[16] Kaye KS, Petty LA, Shorr AF, Zilberberg MD (2019). Current epidemiology, etiology, and burden of acute skin infections in the United States. Clin. Infect. Dis..

[17] Hatoum HT, Akhras KS, Lin SJ (2009). The attributable clinical and economic burden of skin and skin structure infections in hospitalized patients: a matched cohort study. Diagn. Microbiol. Infect. Dis..

[18] Garau J, Ostermann H, Medina J, Avila M, McBride K, Blasi F (2013). Current management of patients hospitalized with complicated skin and soft tissue infections across Europe (2010–2011): assessment of clinical practice patterns and real-life effectiveness of antibiotics from the REACH study. Clin. Microbiol. Infect..

[19] Nathwani D, Eckmann C, Lawson W, Stephens JM, Macahilig C, Solem CT (2014). Pan-European early switch/early discharge opportunities exist for hospitalized patients with methicillin-resistant *Staphylococcus aureus* complicated skin and soft tissue infections. Clin. Microbiol. Infect..

[20] Seaton RA, Johal S, Coia JE, Reid N, Cooper S, Jones BL (2014). Economic evaluation of treatment for MRSA complicated skin and soft tissue infections in Glasgow hospitals. Eur. J. Clin. Microbiol. Infect. Dis..

[21] Matthews PC, Conlon CP, Berendt AR, Kayley J, Jefferies L, Atkins BL (2007). Outpatient parenteral antimicrobial therapy (OPAT): is it safe for selected patients to self-administer at home? A retrospective analysis of a large cohort over 13 years. J. Antimicrob. Chemother..

[22] Tice AD, Rehm SJ, Dalovisio JR, Bradley JS, Martinelli LP, Graham DR (2004). Practice guidelines for outpatient parenteral antimicrobial therapy. IDSA guidelines. Clin. Infect. Dis..

[23] Anastasio PJ, Wolthoff P, Galli A, Fan W (2017). Single-dose oritavancin compared to standard of care IV antibiotics for acute bacterial skin and skin structure infection in the outpatient setting: a retrospective real-world study. Infect. Dis. Ther..

[24] Menarini.: Orbactiv, INN-oritavancin, Summary of Product Characterstics (SmPC), https://www.ema.europa.eu/en/documents/product-information/orbactiv-epar-product-information_en.pdf (accessed 24 Jul 2020)

[25] Nathwani D, Dryden M, Garau J (2016). Early clinical assessment of response to treatment of skin and soft-tissue infections: how can it help clinicians? Perspectives from Europe. Int. J. Antimicrob. Agents..

[26] EMA.: European Medicines Agency; Orbactiv, https://www.ema.europa.eu/en/medicines/human/EPAR/orbactiv (2015, accessed 15 Jul 2020)

[27] Corey GR, Kabler H, Mehra P, Gupta S, Overcash JS, Porwal A (2014). Single-dose oritavancin in the treatment of acute bacterial skin infections. N. Engl. J. Med..

[28] Corey GR, Good S, Jiang H, Moeck G, Wikler M, Green S (2015). Single-dose oritavancin versus 7–10 days of vancomycin in the treatment of gram-positive acute bacterial skin and skin structure infections: the SOLO II noninferiority study. Clin. Infect. Dis..

[29] Thom H, Thompson JC, Scott DA, Halfpenny N, Sulham K, Corey GR (2015). Comparative efficacy of antibiotics for the treatment of acute bacterial skin and skin structure infections (ABSSSI): a systematic review and network meta-analysis. Curr. Med. Res. Opin..

[30] Marwick C, Rae N, Irvine N, Davey P (2012). Prospective study of severity assessment and management of acute medical admissions with skin and soft tissue infection. J. Antimicrob. Chemother..

[31] BNF.: Joint Formulary Committee. British National Formulary. Medicines Complete, https://www.medicinescomplete.com/#/content/bnf/ (2020, accessed 26 Apr 2019)

[32] PSSRU.: Unit costs of health and social care (PSSRU) https://www.pssru.ac.uk/pub/uc/uc2019/services.pdf (2019, accessed 22 Jun 2020)

[33] Curtis LBA (2019). A unit costs of health and social care. Personal social services research unit.

[34] PSSRU.: NHS cost inflation index, Unit Costs of Health and Social Care (PSSRU) 2019, https://www.pssru.ac.uk/pub/uc/uc2019/sources-of-information.pdf (2019, accessed 22 Jun 2020)

[35] NHS.: National Cost Collection: National Schedule of NHS costs—year 2018–2019—NHS trust and NHS foundation trusts, https://improvement.nhs.uk/resources/national-cost-collection/#ncc1819 (2018, accessed 22 Jun 2020)

[36] BNF.: Joint Formulary Committee. Dalbavancin (IV) Correvio UK Ltd. British National Formulary (online) London: BMJ Group and Pharmaceutical Press, https://www.medicinescomplete.com/#/content/bnf/_776052829?hspl=Dalbavancin (2020, accessed 22 Jun 2020)

[37] Ektare V, Khachatryan A, Xue M, Dunne M, Johnson K, Stephens J (2015). Assessing the economic value of avoiding hospital admissions by shifting the management of gram+ acute bacterial skin and skin-structure infections to an outpatient care setting. J. Med. Econ..

[38] AlmarzokyAbuhussain SS, Burak MA, Kohman KN, Jacknin G, Tart SB, Hobbs ALV (2018). Patient preferences for treatment of acute bacterial skin and skin structure infections in the emergency department. BMC Health Serv. Res..

[39] Soriano A, Stefani S, Pletz MW, Menichetti F (2020). Antimicrobial stewardship in patients with acute bacterial skin and skin-structure infections: an international Delphi consensus. J. Glob. Antimicrob. Resist..

[40] Brownell LE, Adamsick ML, McCreary EK, Vanderloo JP, Ernst EJ, Jackson ER (2020). Clinical outcomes and economic impact of oritavancin for gram-positive infections: a single Academic Medical Center Health System Experience. Drugs Real World Outcomes..

[41] Nathwani D (2003). Impact of methicillin-resistant *Staphylococcus aureus* infections on key health economic outcomes: does reducing the length of hospital stay matter?. J. Antimicrob. Chemother..

[42] Tice A (2012). Oritavancin: a new opportunity for outpatient therapy of serious infections. Clin. Infect. Dis..

[43] Lodise TP, Palazzolo C, Reksc K, Packnett E, Redell M (2019). Comparisons of 30-day admission and 30-day total healthcare costs between patients who were treated with oritavancin or vancomycin for a skin infection in the outpatient setting. Open Forum Infect. Dis..

[44] Jensen IS, Wu E, Fan W, Lodise TP, Nicolau DP, Dufour S (2016). Use of oritavancin in moderate-to-severe ABSSSI patients requiring IV antibiotics: a US Payer Budget Impact Analysis. J. Manag. Care Spec. Pharm..

[45] Restelli U, Bonfanti M, Croce D, Grau S, Metallidis S, Moreno Guillén S (2019). Organisational and financial consequences of the early discharge of patients treated for acute bacterial skin and skin structure infection and osteomyelitis in infectious disease departments in Greece, Italy and Spain: a scenario analysis. BMJ Open.

[46] Corey GR, Wilcox MH, Talbot GH, Thye D, Friedland D, Baculik T (2010). CANVAS 1: the first Phase III, randomized, double-blind study evaluating ceftaroline fosamil for the treatment of patients with complicated skin and skin structure infections. J. Antimicrob. Chemother..

[47] Aikawa N, Kusachi S, Mikamo H, Takesue Y, Watanabe S, Tanaka Y (2013). Efficacy and safety of intravenous daptomycin in Japanese patients with skin and soft tissue infections. J. Infect. Chemother..

[48] Itani KM, Dryden MS, Bhattacharyya H, Kunkel MJ, Baruch AM, Weigelt JA (2010). Efficacy and safety of linezolid versus vancomycin for the treatment of complicated skin and soft-tissue infections proven to be caused by methicillin-resistant *Staphylococcus aureus*. Am. J. Surg..

[49] Wilcox MH, Corey GR, Talbot GH, Thye D, Friedland D, Baculik T (2010). CANVAS 2: the second Phase III, randomized, double-blind study evaluating ceftaroline fosamil for the treatment of patients with complicated skin and skin structure infections. J. Antimicrob. Chemother..

[50] Menarini: Menarini data on file: Cost price Oritavancin (2020)

[51] NHS: NHS 2019/20 National Tariff Payment System: proposed national prices and prices for emergency care services. https://www.england.nhs.uk/pay-syst/national-tariff/2019-20-payment-reform-proposals/. Accessed 22 June 2020

[52] Antibiotic Steering Committee, Antibiotic Guidelines (Adult): Skin and Soft Tissue Infections (SSTI). Salford Royal NHS Foundation Trust (2014)

